# Persistent Legionnaires’ Disease and Associated Antibiotic Treatment Engender a Highly Disturbed Pulmonary Microbiome Enriched in Opportunistic Microorganisms

**DOI:** 10.1128/mBio.00889-20

**Published:** 2020-05-19

**Authors:** Ana Elena Pérez-Cobas, Christophe Ginevra, Christophe Rusniok, Sophie Jarraud, Carmen Buchrieser

**Affiliations:** a Institut Pasteur, Biologie des Bactéries Intracellulaires, Paris, France; b CNRS UMR 3525, Paris, France; c CIRI, International Center for Infectiology Research, Legionella pathogenesis Team, Université de Lyon, Lyon, France; d Inserm, U1111, Lyon, France; e Ecole Normale Supérieure de Lyon, Lyon, France; f Université Lyon 1, Centre International de Recherche en Infectiologie, Lyon, France; g CNRS, UMR5308, Lyon, France; h Hospices Civils de Lyon, Centre National de Référence des Légionelles, Lyon, France; University of Illinois at Chicago

**Keywords:** *Legionella pneumophila*, antibiotic resistance, pneumonia, pulmonary microbiome

## Abstract

Despite the importance of pneumonia to public health, little is known about the composition of the lung microbiome during infectious diseases, such as pneumonia, and how it evolves during antibiotic therapy. To study the possible relation of the pulmonary microbiome to the severity and outcome of this respiratory disease, we analyzed the dynamics of the pathogen and the human lung microbiome during persistent infections caused by the bacterium *Legionella pneumophila* and their evolution during antimicrobial treatment. We collected 10 bronchoalveolar lavage fluid samples from three patients during long-term hospitalization due to pneumonia and performed a unique longitudinal study of the interkingdom microbiome, analyzing the samples for presence of bacteria, archaea, fungi, and protozoa by high-throughput Illumina sequencing of marker genes. The lung microbiome of the patients was characterized by a strong predominance of the pathogen, a low diversity of the bacterial fraction, and an increased presence of opportunistic microorganisms. The fungal fraction was more stable than the bacterial fraction. During long-term treatment, no genomic changes or antibiotic resistance-associated mutations that could explain the persistent infection occurred, according to whole-genome sequencing analyses of the pathogen. After antibiotic treatment, the microbiome did not recover rapidly but was mainly constituted of antibiotic-resistant species and enriched in bacteria, archaea, fungi, or protozoa associated with pathogenicity. The lung microbiome seems to contribute to nonresolving *Legionella* pneumonia, as it is strongly disturbed during infection and enriched in opportunistic and/or antibiotic-resistant bacteria and microorganisms, including fungi, archaea, and protozoa that are often associated with infections.

## Abstract (precis)

The composition and dynamics of the lung microbiome during pneumonia are not known, although the lung microbiome might influence the severity and outcome of this infectious disease, similar to what was shown for the microbiome at other body sites. Here we report the findings of a comprehensive analysis of the lung microbiome composition of three patients with long-term pneumonia due to L. pneumophila and its evolution during antibiotic treatment. This work adds to our understanding of how the microbiome changes during disease and antibiotic treatment and points to microorganisms and their interactions that might be beneficial. In addition to bacteria and fungi, our analyses included archaea and eukaryotes (protozoa), showing that both are present in the pulmonary microbiota and that they might also play a role in the response to the microbiome disturbance.

## INTRODUCTION

Historically, the lower airways were thought to be sterile unless they were infected. However, the culture-independent and high-throughput sequencing techniques that have been developed have revealed that the respiratory tract, like almost every mucosal surface in the human body, harbors a distinct microbiome composed of bacteria, fungi, and viruses (1–3). In 2016, the bacterial composition of the healthy lung was deeply characterized for the first time by analyzing bronchoalveolar lavage (BAL) fluid samples from healthy individuals. Two types of microbiome profiles (termed “pneumotypes”) were described (4). One of the pneumotypes, named supraglottic predominant taxa (SPT), was characterized by a high bacterial load and the presence of anaerobes, such as *Prevotella* and *Veillonella*, belonging to the phyla *Bacteroides* and *Firmicutes*, respectively, whereas the second pneumotype was called the background predominant taxa (BPT), as it presented a low biomass and consisted of bacteria that mainly belonged to the phylum *Proteobacteria*, such as *Acidocella* or *Pseudomonas*. Although analysis of the lung microbiome is a relatively new field and little is known about its role in human health, it was suggested that the lung microbiome participates in immune system functions, including immune cell maturation and inflammatory responses (2, 5). Segal and colleagues proposed that bacteria, such as *Prevotella* and *Veillonella*, that have been commonly identified in the lungs of healthy individuals or their products participate in the regulation of inflammation and are linked to the activation of the lung mucosal Th17 response (4).

The fungal microbiome of a healthy lung is mainly composed of environmental fungi belonging to the phyla Ascomycota and Basidiomycota, such as the Davidiellaceae, *Eurotium*, *Eremothecium*, and *Cladosporium*, while during disease, a higher abundance of pathogenic species belonging to the genera *Aspergillus*, *Malassezia*, or *Candida* is present (6). Commensal fungi, like bacteria, seem to participate in immune system stimulation, the inflammatory response, and protection against pathogens (5, 6). In contrast to the bacterial and fungal composition of the lung, which has started to be investigated better, the presence of eukaryotes, archaea, or viruses has been investigated very little.

To date, the majority of studies analyzing the lung microbiome in disease have focused on cystic fibrosis (7–9), asthma (10, 11), or chronic obstructive pulmonary disease (COPD) (12–14). These studies have shown that lung microbial dysbiosis is generally characterized by a microbiome enriched in pathogenic and opportunistic bacteria (such as certain *Gammaproteobacteria*) with a high biomass due to the overgrowth of pathogens and a low diversity because of the displacement of the normal members of the community (2, 5).

Among infectious diseases, acute lower respiratory tract infections are the leading cause of morbidity and mortality worldwide. Among these, pneumonia represents a clinical and economic burden and a major public health problem, particularly for children and the elderly. The bacterium Legionella pneumophila is one of the human pathogens (15) that can cause a severe pneumonia, called Legionnaires’ disease, which can be fatal in about 8% of the cases, despite timely and adequate therapy (16). However, Legionnaires’ disease is characterized by clinical polymorphism and variable severity. For example, in France, 98% of recognized patients with Legionnaires’ disease are hospitalized and 40% require admission to an intensive care unit (ICU), where the mortality rate remains high (>25% in ICUs) (17). Recently, Mizrahi and collaborators characterized the microbiome of nine sputum samples from patients with L. pneumophila infection, reporting a high abundance of *Streptococcus* bacteria and a low abundance of L. pneumophila bacteria (18). However, lung samples, like pulmonary lavage or tracheal aspirate samples, have not been analyzed yet.

We report the findings of a comprehensive analysis, performed by using high-throughput sequencing of marker genes, of the interkingdom (bacteria, archaea, and eukaryotes) lung microbiome of three patients with long-term pneumonia due to L. pneumophila and its evolution during antibiotic therapy.

## RESULTS

### Lung samples that originated from patients and healthy individuals.

**FIG 1:**
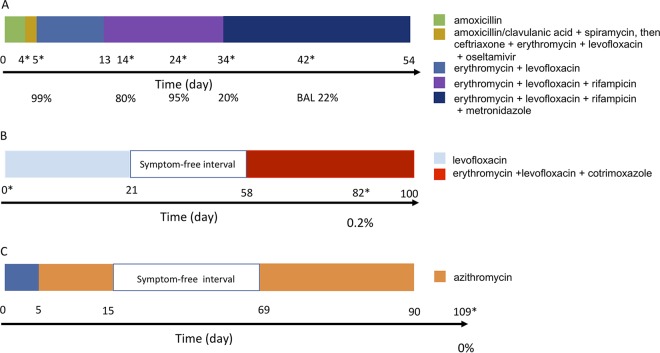
History of the antibiotic treatments of the patients analyzed here. (A) Patient A; (B) patient B; (C) patient C. The colors indicate the antibiotic treatment, and the length of the colored block indicates the duration of treatment with each antibiotic. The numbers below the colored blocks indicate the days on which a sample was taken. The days on which BAL fluid samples were collected for analysis of the microbiome are marked with an asterisk. The percentage given below the timeline indicates the relative abundance of *Legionella* in the sample, as determined by qPCR.

The medical histories of the three patients included in this study have been described as a case series of slowly resolving and nonresolving Legionnaires’ disease (19), and a detailed description of patient A’s clinical course has also been described (18). This retrospective research, conducted by the French National Reference Center for *Legionella* (2013 to 2017), reported several Legionnaires’ disease cases with persistent clinical symptoms, computed tomography (CT) scan abnormalities, and *Legionella* detection in lower respiratory tract specimens by culture and/or real-time (RT) PCR (18). Here, we analyzed the lung microbiome composition of three of the patients included in this previous study (19). Briefly, patient A (patient 3 in reference 19) was a 28-year-old immunocompetent man admitted to the hospital due to severe community-acquired pneumonia (CAP). For 5 days, he was treated with amoxicillin, as a Streptococcus pneumoniae infection was suspected. As the pneumonia got worse, this treatment was enlarged with amoxicillin-clavulanic acid and spiramycin and, later, with ceftriaxone, erythromycin, levofloxacin, and oseltamivir. Only at day 5 was L. pneumophila identified as the causative agent, and the treatment was switched to a combination of erythromycin and levofloxacin (19, 20) (Fig. 1A). Due to a suspicion of the presence of a lung abscess according to a thoracic computed tomography (CT) scan, rifampin was added at day 13. A second thoracic CT scan revealed a voluminous lung abscess at day 34, and Fusobacterium nucleatum was identified. Thus, metronidazole was added for 20 more days. The abscess was resected on day 42, and the patient recovered fully. We analyzed the microbiome composition of the BAL fluid samples taken at days 5, 14, 24, 34, and 42 postadmission as well as that of one sputum sample taken at day 4 postadmission and a biopsy sample taken during lung abscess removal at day 42 (Fig. 1A).

Patient B (patient 8 in reference 19), a 69-year-old immunocompromised woman, was hospitalized for *Legionella*-associated pneumonia and treated for 21 days with levofloxacin. After she had been free of symptoms for 37 days, she was rehospitalized for bilateral pulmonary consolidations and pleural effusion. A recurrent *Legionella* pneumonia was confirmed, and she was treated for 6 weeks with erythromycin, levofloxacin, and co-trimoxazole for a concomitant pneumocystosis (19). A sample was taken at the onset of treatment (day 0) and after 2.5 months (day 82) (Fig. 1B).

Patient C (patient 10 in reference 19), a 76-year-old immunocompromised man, was sampled 109 days after the onset of the treatment, when the patient had fully recovered. The sample was taken 19 days after the end of antibiotic therapy. This BAL fluid sample (named sample C109) was negative for L. pneumophila, as confirmed by PCR (19) (Fig. 1C).

To compare the microbiomes of lungs with pneumonia with those of healthy lungs, we retrieved and reanalyzed the data for the lung microbiomes of 49 healthy individuals in our pipeline (4). The published data were obtained from BAL fluid samples and were characterized by sequencing the 16S rRNA gene (bacterial microbiome) (4). The persons defined to be healthy in this study did not present underlying lung diseases; had not been treated with antibiotics or steroids in the 3 months prior to sampling; had no cardiovascular, renal, or liver disease or diabetes mellitus; and did not have heavy alcohol use (more than six beers daily). They also did not present respiratory symptoms (cough, wheezing, or shortness of breath) before bronchoscopy (4).

### BAL fluid and sputum samples differ in microbiome composition.

The comparison of the BAL fluid sample taken on day 5 and the sputum sample taken on day 4 (patient A) identified *Streptococcus* (43%), *Prevotella* (13%), and *Gemella* (13%) to be the most abundant bacterial genera in the sputum sample, while *Legionella* represented only 3% of the total relative abundance (see Fig. S1A in the supplemental material). Interestingly, the fungal microbiome of both the sputum and the BAL fluid samples showed a high abundance of Ascomycota (40%) (Fig. S1B). In contrast, the BAL fluid sample showed a lower diversity and operational taxonomic unit (OTU) richness than the sputum sample, and more than 99% of the sequences belonged to *Legionella* (Fig. S1C). This reveals that, during pneumonia, the abundance of *Legionella* compared to that of other bacteria is low in sputum, while *Legionella* is very abundant in the BAL fluid samples, which represent the lung. Thus, the analysis of sputum samples and the analysis of BAL fluid samples are two approaches that might be coupled in analyzing the lung microbiome to have the best picture of the microbiomes in the upper and lower respiratory tract during disease.

10.1128/mBio.00889-20.5 FIG S1 Comparison of the microbiome composition of the BAL fluid and sputum samples and the bacterial alpha diversity of the microbiomes of patients A and B. For each sample, the estimated values and the error bars are shown. Download FIG S1, TIF file, 2.5 MB. Copyright © 2020 Pérez-Cobas et al. 2020 Pérez-Cobas et al. This content is distributed under the terms of the Creative Commons Attribution 4.0 International license.

### Legionella pneumophila strains isolated during infection and antimicrobial therapy show no specific resistance profile.

In a previous study, one L. pneumophila strain isolated at the start of hospitalization and one isolated at the end of hospitalization were analyzed (19). They did not exhibit phenotypic antibiotic resistance or have genomic mutations that could be related to antibiotic resistance (19). To follow the evolution of the pathogen in more detail, in the present study we performed whole-genome sequencing of 15 intermediate isolates recovered over the entire hospitalization period from patient A and 9 isolates (4 early isolates and 5 late isolates) recovered from patient B. Mapping of the corresponding genomes to those of the earliest isolates and a search for genes related to antibiotic resistance did not identify any genomic changes between the different isolates for either patient. Thus, L. pneumophila does not seem to evolve fast under antibiotic pressure, in line with our findings presented in a previous report, where we estimated a very low evolutionary rate of 0.71 single nucleotide polymorphism per genome per year for L. pneumophila strains (21). To further corroborate these results, long-read sequencing was performed for the first and the last isolates from patient A; however, this approach also did not identify any chromosomal rearrangements or recombination events, suggesting that the resistance of these isolates must be due to factors other than changes in their genome sequences.

### Antibiotic treatment leads to strong perturbations and the slow recovery of a healthy microbiome.

**FIG 2:**
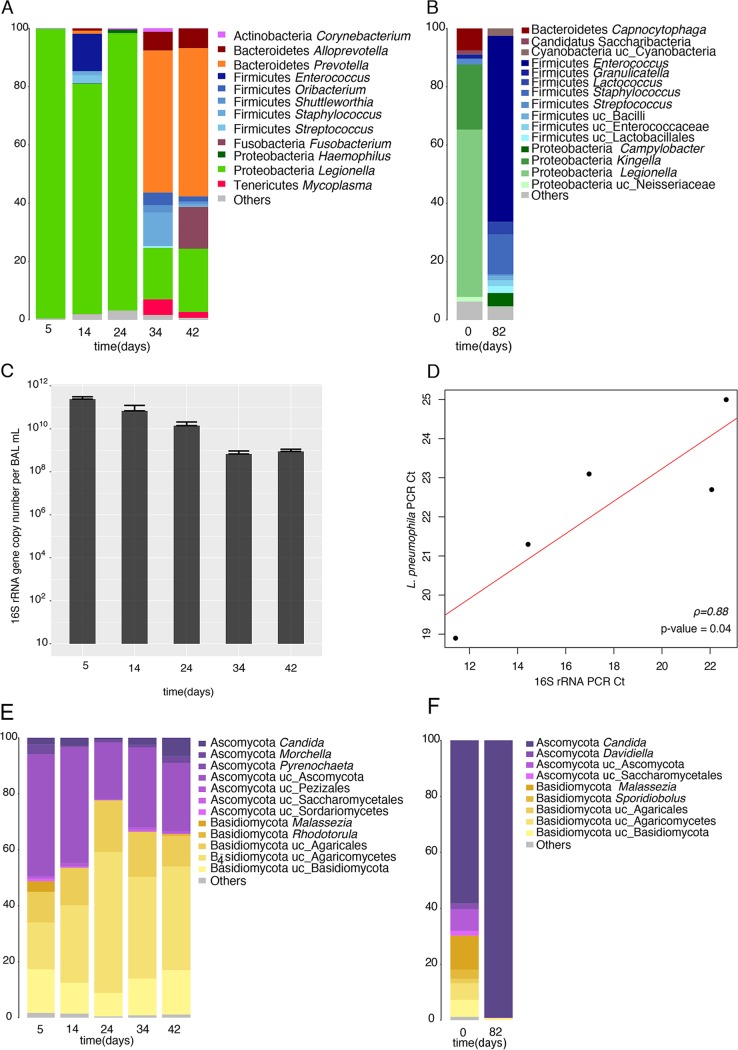
Microbiome and mycobiome composition of the BAL fluid samples. (A and B) Bacterial composition of the BAL fluid samples from patient A (A) and patient B (B). The numbers on the *y* axes are percent abundance. (C) Number of total bacteria (patient A) estimated by qPCR of the 16S rRNA gene. (D) Correlation of the number of bacteria (*C_T_* of the 16S rRNA gene determined by qPCR) with the amount of *Legionella* (*C_T_* of the *mip* gene determined by qPCR) (patient A). The *C_T_* values for the *mip* gene were obtained from a clinical case study (20). (E and F) Fungal composition of the BAL fluid samples from patient A (E) and patient B (F).

*Legionella* dominated the bacterial composition of the BAL fluid samples of patients A and B during the first days of antibiotic treatment. For patient A, at the time of diagnosis, *Legionella* represented 99% of the bacteria identified in the lungs, and the *Legionella* bacteria stayed dominant until day 24, when they still represented 95% of the bacteria (Fig. 2A). Similarly, the microbiome of patient B was mainly composed of *Legionella*, representing 57% of the diversity at the beginning of treatment (Fig. 2B). After long-term antibiotic therapy, a substantial change in the microbiome composition of patients A (Fig. 2A) and B (Fig. 2B) occurred. Both patients showed a marked decrease in the relative abundance of *Legionella* (20% for patient A and 0.2% for patient B), indicating that the antibiotic treatment was efficient. Furthermore, the presence of bacteria commonly found in the lung microbiome of healthy individuals (5), such as *Prevotella* (from less than 1% during the first 24 days of therapy to about 50% at day 34) and *Staphylococcus* (11%) for patient A and *Enterococcus* (64%) and *Staphylococcus* (14%) for patient B, increased. Indeed, certain species of the genus *Prevotella* are resistant to different classes of antibiotics, including macrolides, which were included in the treatment (22). Furthermore, *Prevotella* was described to be a predominant taxon in healthy microbiomes (4), suggesting that the microbiome started to recover. The bacterial diversity of the microbiome of patient A collapsed due to the antibiotic treatment at about day 24, reaching the lowest values for all estimated metrics (Fig. S1C). For both patients, the community richness was higher at the end of the infection (Fig. S1C and D).

Analysis of the evolution of the bacterial load of patient A showed a decrease in biomass during antibiotic treatment (Fig. 2C). During the first days of therapy, the load was very high (10^10^ to 10^12^ copies of the 16S rRNA gene), but after day 24 the load decreased significantly to 10^8^ to 10^9^ copies, probably due to the addition of rifampin at day 13 and/or to the time needed for the activity of the combination of a fluoroquinolone plus a macrolide (*P* = 0.002). The Pearson correlation test between the threshold cycle (*C_T_*) values of L. pneumophila and the total biomass (16S rRNA data) pointed to a significant positive correlation between the two (*P* = 0.04) (Fig. 2D). Thus, the lower biomass can be partially explained by the decrease in pathogen abundance due to therapy.

### During pneumonia and antibiotic therapy, the fungal microbiome is less disturbed.

The fungal composition of the BAL fluid samples of patients A (Fig. 2E) and B (Fig. 2F) was mainly composed of two phyla, Basidiomycota and Ascomycota. These are reported to be the most abundant phyla in the human respiratory tract (6). However, most of the OTUs could be classified only at the level of the phylum, suggesting that the true diversity of the fungi awaits characterization. The main genus identified was *Candida* (for patient A, 0.8 to 7%; for patient B, 65 to 99%). Despite the higher homogeneity of the fungal composition than the bacterial one, the richness and diversity of patient A decreased like those of the bacteria through the antibiotic treatment and started to recover by day 34 of treatment (Fig. S2A). For patient B, after several weeks of antibiotic treatment, the genus *Candida* constituted about 99% of the fungal microbiome composition, explaining why the diversity metrics were lower after therapy (Fig. S2B). The few studies that have analyzed the fungal microbiome have reported that in healthy people it consists predominantly of environmental organisms, such as *Aspergillus* or *Cladosporium* (23). This composition is clearly different from that of the fungal microbiome of the patients determined in the present study. The changes in the mycobiome during bacterial infection and antibiotic treatment suggest that the fungal and the bacterial communities interact ecologically in the lungs (i.e., cooperation, competition) and with the host immune system. Furthermore, infection and the associated antibiotic treatment change the healthy lung microbiome considerably and allow *Candida* species to occupy the niche, which is disturbed by the pathogen and the antibiotic treatment.

10.1128/mBio.00889-20.6 FIG S2 Fungal alpha diversity of the microbiomes of patients A and B. For each sample, the estimated values and the error bars are shown. Download FIG S2, TIF file, 2.7 MB. Copyright © 2020 Pérez-Cobas et al. 2020 Pérez-Cobas et al. This content is distributed under the terms of the Creative Commons Attribution 4.0 International license.

### Enrichment of the lung abscess with pathogenic microorganisms.

**FIG 3:**
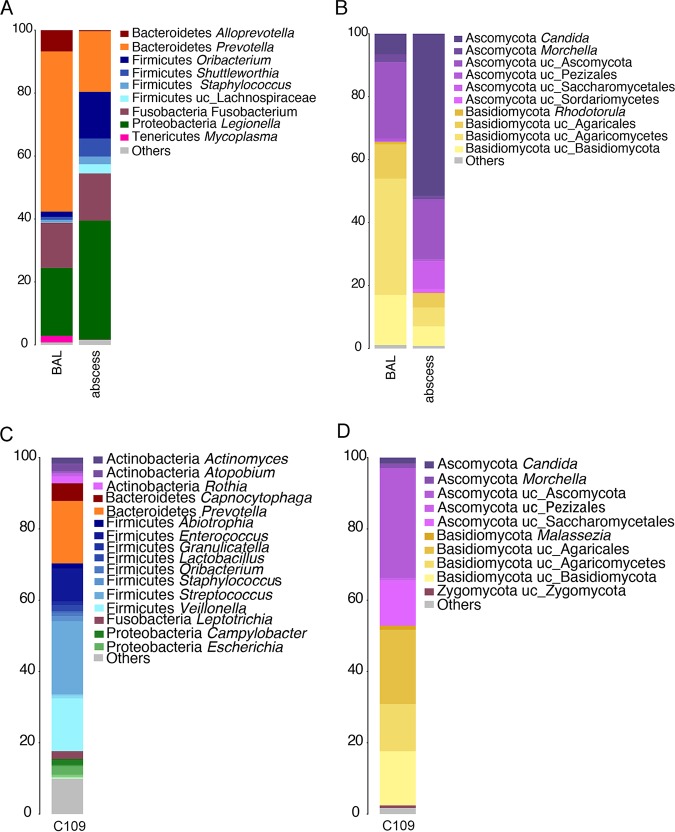
Comparison of the microbiome composition of the BAL fluid and abscess samples and the microbiome composition of patient C. (A) Bacterial composition. The taxonomy is based on the RDP. (B) Fungal composition. The taxonomy is based on the Warcup ITS training set. (C and D) Bacterial composition (C) and fungal composition (D) at 19 days after treatment. The numbers on the *y* axes are percent abundance.

The microbial composition of the lung abscess was determined to be *Legionella* (38%), *Prevotella* (19%), *Fusobacterium* (15%), and *Oribacterium* (15%) (Fig. 3A). As expected, *Legionella* was more abundant in the lung abscess samples than in the BAL fluid samples. Also, anaerobic bacteria, such as *Fusobacterium*, *Oribacterium*, and *Shuttleworthia*, showed a higher abundance in the abscess, probably because the environment is more anoxic than that in other areas of the lungs. However, the diversity was lower in the abscess than in the BAL fluid samples (Fig. S1C). The fungal composition of the abscess (Fig. 3B) was dominated by *Candida* (50%) and other Ascomycota (20%), revealing enrichment of this phylum. The OTU richness was higher in the lung abscess than in the BAL fluid samples. However, the Shannon diversity index was lower, as only a few fungi were dominant (Fig. S2A).

### After long-term antibiotic treatment, the microbiome is enriched in *Firmicutes*.

To analyze the recovery of the microbiome after infection and long-term antibiotic treatment, we characterized the lung microbiome composition of patient C. The bacterial microbiome was enriched in *Firmicutes*, such as *Streptococcus* (20%), *Veillonella* (15%), and *Enterococcus* (10%), followed by *Bacteroidetes*: *Prevotella* (17%) (Fig. 3C). *Prevotella* and *Veillonella* are present in the lungs of healthy people, suggesting that the lung microbiome of this patient was in the restoration process (4). The fungal microbiome was mainly composed of Ascomycota (40%) and Basidiomycota (38%) (Fig. 3D). Similar to the samples from patient B, the evenness (Shannon diversity index = 4.9) and OTU richness (Chao 1 richness estimator = 2,348, number of OTUs = 990) after treatment for the samples from patient C were higher than those for the samples from patients A and B during infection.

### Archaea are part of the lung microbiota.

As it has been reported that archaea are present at all body sites, including the nose and lung (24), we analyzed our BAL fluid samples for archaea (Table S1A). Indeed, *Methanobrevibacter*, representing more than 50% of the archaeal diversity, was present in all samples (Table S1B). Patients A and C also carried other unclassified *Euryarchaeota* and *Thermoprotei* (*Crenarchaeota*), while in patient C, only archaea belonging to the genus *Methanobrevibacter* were identified. The closest species of the most abundant OTUs was Methanobrevibacter smithii, which was surprising, as it is described to be an anaerobic methane-producing archaeon. Thus, it is possible that there are anaerobic niches in the lung environment or that other bacteria present in the lung help this anaerobic archaeon to grow. Indeed, the aerobic culture of methanogenic archaea without an external source of hydrogen has been reported when Bacteroides thetaiotaomicron, which produces hydrogen, is present (25). Interestingly, methanogenic archaea have been associated with different diseases, suggesting that they may contribute to the disease under specific conditions and, thus, perhaps also to pneumonia (24).

10.1128/mBio.00889-20.2 TABLE S1 (A) Proportion of reads classified as bacteria or archaea for each sample. (B) Relative abundance of archaea present in the BAL fluid samples. (C) Relative abundance of protozoa in the BAL fluid samples. Download Table S1, DOCX file, 0.02 MB. Copyright © 2020 Pérez-Cobas et al. 2020 Pérez-Cobas et al. This content is distributed under the terms of the Creative Commons Attribution 4.0 International license.

### *Legionella*-related amoebae were identified in the human lungs.

Environmental amoebae are the reservoir of *Legionella*, and amoebae infected with *Legionella* might be a vehicle of transmission. Inhaled infected protozoa may serve as cofactors in the pathogenesis of pulmonary disease (26). Thus, we analyzed the samples for the presence of the genus *Acanthamoeba* and the class Heterolobosea. A large proportion was identified to be unclassified eukaryotes, suggesting that considerable eukaryotic diversity is present in the lungs and remains to be described. Indeed, we identified Acanthamoeba castellanii (Table S1C) and Trichomonas tenax (Table S2), a protozoan commonly found in the human oral cavity but rarely associated with pulmonary infections (27). Acanthamoeba castellanii is an aquatic protozoan that is a natural host of *Legionella* and that may be important in the transmission of this pathogen, but it also could be part of a resident protozoan community in the lungs. However, further studies using various genus-specific primers and samples from healthy individuals need to be undertaken to answer this question.

10.1128/mBio.00889-20.3 TABLE S2 Relative abundance of eukaryotic sequences in the BAL fluid samples. Download Table S2, DOCX file, 0.02 MB. Copyright © 2020 Pérez-Cobas et al. 2020 Pérez-Cobas et al. This content is distributed under the terms of the Creative Commons Attribution 4.0 International license.

### The lung microbiome during infection and antimicrobial therapy is significantly different from a healthy microbiome.

**FIG 4:**
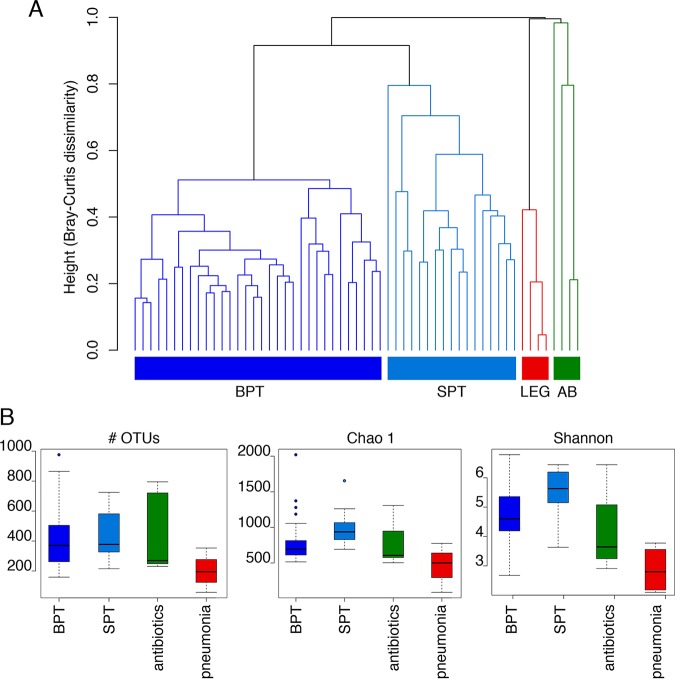
Comparison of the bacterial composition of healthy pneumotypes and *Legionella*-infected and antibiotic-treated BAL fluid samples. (A) Hierarchical clustering of all samples based on the bacterial composition. A hierarchical clustering analysis based on Bray-Curtis dissimilarity was used as a distance method. BPT (*n* = 32), background predominant taxa; SPT (*n* = 17), supraglottic predominant taxa; LEG (*n* = 4), BAL fluid samples from *Legionella*-infected patients (patient A samples were taken at days 5, 14, and 24; the patient B sample was taken at day 0); AB (*n* = 4), BAL fluid samples after antibiotic treatment (patient A samples were taken at days 34 and 42; the patient B sample was taken at day 82; the patient C sample was sample C109). (B) Comparison of the diversity between the two healthy pneumotypes (*n* = 49) and the pneumonia (*n* = 4) and antibiotic-treated (*n* = 4) samples. The diversity metrics Chao 1 richness estimator, the number of OTUs, and the Shannon diversity index were estimated for the three groups. Each box plot represents the distribution of values, including the median, minimum, maximum, first and third quartiles, and outliers.

To better understand the differences between the lung microbiome of healthy individuals and the one influenced by infection and antibiotic treatment, we compared the healthy microbiomes reported by Segal and colleagues in 2016 (4) with the microbiomes in our patients’ samples (Fig. S3). They analyzed bronchoalveolar lavage (BAL) fluid samples from healthy individuals. Two types of microbiome profiles (termed pneumotypes) were described (4). One of the pneumotypes, named supraglottic predominant taxa (SPT), was characterized by a high bacterial load and the presence of anaerobes, such as *Prevotella* and *Veillonella*, belonging to the phyla *Bacteroidetes* and *Firmicutes*, respectively. In contrast, the second pneumotype was called the background predominant taxa (BPT), as it presented a low biomass and consisted of bacteria that belonged mainly to the phylum *Proteobacteria*, such as *Pseudomonas* and *Acidocella*. The compositions of the microbiomes of healthy people and the ones of the patients were significantly different (adonis test, *P* = 0.004975). Clustering analyses grouped the microbiomes of the pneumonia patients separately from those of the healthy ones (Fig. 4A). Furthermore, the samples containing a high abundance of *Legionella* (patient A samples taken at days 5, 14, and 24 and the patient B sample taken at day 0) clustered together, while the microbiomes of samples taken after antibiotic treatment (patient A samples taken at days 34 and 42, the patient B sample taken at day 82, and patient C sample C109) were different.

10.1128/mBio.00889-20.7 FIG S3 Lung microbiome composition (SPT and BPT) of samples from healthy subjects and patients with pneumonia. Download FIG S3, TIF file, 2.7 MB. Copyright © 2020 Pérez-Cobas et al. 2020 Pérez-Cobas et al. This content is distributed under the terms of the Creative Commons Attribution 4.0 International license.

When the healthy microbiome was statistically compared with that of the samples from the patients (disrupted microbiome), 34 families (most of them *Proteobacteria*) were significantly more abundant in the microbiomes of the healthy people (Table S3). The microbiomes of the pneumonia patients showed a high abundance of only five families: *Legionellaceae*, *Staphylococcaceae*, *Streptococcaceae*, *Propionibacteriaceae*, and *Corynebacteriaceae*. The most abundant OTUs were classified as L. pneumophila, a *Staphylococcus* sp., Streptococcus sanguinis, Cutibacterium acnes, and a *Corynebacterium* sp., respectively. Furthermore, diversity and richness were significantly lower in the samples from patients than in the ones from healthy people (*P* < 0.05) (Fig. 4B). The healthy microbiome showed a higher diversity and richness than the antibiotic-treated one (*P* > 0.05), indicating an intermediate state of diversity after therapy.

10.1128/mBio.00889-20.4 TABLE S3 Bacterial families identified to have statistically significantly different abundances between healthy subject and patient samples. Download Table S3, DOCX file, 0.02 MB. Copyright © 2020 Pérez-Cobas et al. 2020 Pérez-Cobas et al. This content is distributed under the terms of the Creative Commons Attribution 4.0 International license.

### Ecological interactions between bacterial and fungal communities in the human lungs.

The parallel changes in bacterial and fungal diversity observed in patient A led us to analyze possible interactions between the two microbial communities. To get insight into the ecology of these communities and their possible interactions, we used the Wilcoxon signed-rank test and compared the diversity metric distributions of both communities. Then, we established a correlation network for the microbiome and mycobiome and networks that were specific to each domain to identify putative associations between members of each community. Indeed, putative bacterium-bacterium, bacterium-fungus, and fungus-fungus interactions were identified. These interactions might be critical for the disease outcome and recovery after the cessation of antibiotic treatment (for details, see Text S1 and Fig. S4 and S5).

10.1128/mBio.00889-20.1 TEXT S1 Extended Material and Methods. Download Text S1, DOCX file, 0.03 MB. Copyright © 2020 Pérez-Cobas et al. 2020 Pérez-Cobas et al. This content is distributed under the terms of the Creative Commons Attribution 4.0 International license.

10.1128/mBio.00889-20.8 FIG S4 Correlation of the diversity of the bacterial and fungal communities in the lungs. Download FIG S4, TIF file, 2.7 MB. Copyright © 2020 Pérez-Cobas et al. 2020 Pérez-Cobas et al. This content is distributed under the terms of the Creative Commons Attribution 4.0 International license.

10.1128/mBio.00889-20.9 FIG S5 Correlation networks of taxa in the lung microbiome during pneumonia. Download FIG S5, TIF file, 2.8 MB. Copyright © 2020 Pérez-Cobas et al. 2020 Pérez-Cobas et al. This content is distributed under the terms of the Creative Commons Attribution 4.0 International license.

## DISCUSSION

This unique and comprehensive, longitudinal analysis of the interkingdom lung microbiome of patients suffering for several months from pneumonia due to L. pneumophila revealed that the pathogen strongly dominates the bacterial microbiome and that fungi, archaea, and eukaryotes are also present. The comparison with healthy lung microbiomes showed that the infection engenders a highly disturbed microbial community. During the first stage of the disease, the pathogen is highly abundant, outcompeting other bacteria in the microbiome, explaining the low diversity that we observed. Along this line, studies of lung infection by Pseudomonas aeruginosa or Mycoplasma pneumoniae showed a dominance of the pathogens in the microbiome (28–30). A low microbiome diversity has also been described for pneumonia in patients with HIV infection, cystic fibrosis (5, 28), or pulmonary tuberculosis (31, 32). Despite a low microbial diversity, absolute quantification of the bacteria present in the BAL fluid samples analyzed revealed a very high bacterial biomass during infection (up to 10^11^ bacteria per ml of BAL fluid) due to the high abundance of the pathogen. Thus, although the possibility of contamination derived from bronchoscopy and the saline solution cannot be excluded in this study, such contamination, if present, would significantly affect the results only for BAL fluid samples with lower bacterial loads (less than 8E+04 16S rRNA copies/ml), which was not the case in our study (33).

Our detailed analyses of the genomes of different isolates of the pathogen collected during the entire hospitalization period suggested that the persistence of the infection was not due to changes in the DNA sequence leading to the antibiotic resistance of the bacterium, in agreement with phenotypic and genomic data published previously (19). Thus, the increased tolerance toward antibiotics observed *in vivo* might have been due to the upregulation of antibiotic efflux pumps or to the formation of L. pneumophila persisters (34, 35). Furthermore, other factors might have influenced the weak response of these individuals, including the presence of specific microorganisms from the lung community and their ecological interactions. The overabundance of opportunistic bacteria during infection and after antibiotic therapy indicates that they might contribute to the slower response to the disease, as certain opportunistic species may take advantage of the host inflammatory responses induced by infection. For example, we identified Streptococcus sanguinis to be associated with pneumonia caused by *Legionella*. Indeed, it has been shown that the generation of intra-alveolar catecholamines and inflammatory cytokines during lung infections alters the microbial growth conditions, thereby favoring specific bacterial groups, including *Streptococcus* (36). Also, the presence of S. sanguinis was identified in community-acquired streptococcal pneumonia (37). Furthermore, a retrospective study identified S. sanguinis to be associated with lung abscess development (38). In line with these reports, we identified S. sanguinis to be predominant in the examined abscess sample (40% of the relative abundance). Our results provide important information on the microbiome changes in pneumonia patients; however, it needs to be taken into account that the individuals analyzed here represent a particular group among Legionnaires’ disease patients, as they suffered from a long-term infection and an inadequate response to treatment. Thus, further studies of the lung microbiome composition of Legionnaires’ disease patients, including standard cases, will clarify whether this trend is maintained in all patients.

Very little is known about the role of fungi in lung health and the response to infection. Here, we show that the mycobiome followed more stable dynamics than the microbiome during infection and antibiotic treatment, with Ascomycota and Basidiomycota being the most abundant phyla. However, the antibiotics promoted the presence of *Candida*, which is often associated with extensive antibiotic usage in hospitals (39). Interestingly, many differences between the mycobiomes of the three patients analyzed here and those of healthy individuals investigated in other studies were observed. In the fungal microbiome of the pneumonia patients, genera such as *Candida* or *Malassezia* were present, but the healthy lung microbiome was described as consisting of environmental fungi, such as *Aspergillus* and *Cladosporium* (23). Indeed, *Candida* species are known opportunistic pathogens that have been associated with different lung conditions, including cystic fibrosis (40) and lung transplantation (41). Moreover, *Malassezia* has been connected to asthma (42). Thus, these two genera seem to be typically present in diseased lungs. Furthermore, the identification of correlations between the diversity and the composition of the bacterial and fungal communities present in the lungs of the individuals analyzed here may suggest an ecological relationship between the two communities that could be key for restoration of the microbiome after the cessation of disease and antimicrobial therapy.

Interestingly, archaea may also play a role in disease severity or outcome, as suggested by the presence of Methanobrevibacter smithii in all the patients. Methanogenic archaea have been associated with disease under specific conditions and, thus, perhaps also during pneumonia (24). This could be critical, since archaea are generally resistant to most antibiotics (43). Furthermore, we showed here that amoebae and other protozoans are present in the lungs. This might partly be because *Legionella*-infected amoebae were inhaled, but we identified different protozoa; thus, it seems that the lung microbiota also contains a community of protozoa. *Acanthamoeba* (identified in all the patients) is a natural host of *Legionella* that possibly plays a role in disease transmission. Interestingly, animal studies showed that certain *Acanthamoeba* species, such as A. castellanii or A. polyphaga, might also induce direct damage to the pulmonary parenchyma by causing pneumonitis (44). Furthermore, Trichomonas tenax, which was identified in the lung samples analyzed here, has also been described in pulmonary pathologies (27). Hence, environmental protozoa might play a role in the transmission and the severity of the pathology. The presence of amoeba in all patients analyzed here also supports the possibility that resident protozoa might be present in the human lung microbiome, as is the case in the gut (45) or the oral cavity (46). Indeed, free-living amoebae are commonly found in our air (47), and the isolation of amoebae from nasal passages and the pharynxes of humans has been reported (48). Whether the identified protozoa are part of the healthy microbiome or are particularly present in patients with *Legionella*-associated pneumonia or other diseases needs to be studied further. Thus, we show here that interkingdom interactions also need to be considered in the outcome of the infection and antibiotic therapies. Further large-scale studies are needed to identify markers of a healthy lung microbiome and to understand the time that it takes for a patient to recover a fully healthy lung microbiome.

## MATERIALS AND METHODS

### Sample collection, *Legionella* detection, and DNA extraction.

Bronchoalveolar lavage (BAL) fluid samples (collected at 5, 14, 24, 34, and 42 days after hospital admission) and a sputum specimen (collected at day 4 after hospital admission) were collected from patient A, a sputum specimen was collected from patient B (at days 0 and 82 after hospital admission), and a sputum specimen was collected from patient C (at 109 days after hospital admission or 19 days after the end of therapy) during the hospitalization period. Furthermore, a biopsy specimen of the lung abscess of patient A (collected on day 42) was included (Fig. 1). All samples were stored at −80°C. DNA was extracted from 1 ml of BAL fluid using a PowerSoil DNA isolation kit (Mobio) following the manufacturer’s instructions. The presence of L. pneumophila in the samples was detected by diagnostic PCR using the primers and probes of the R-DiaLeg kit (Diagenode, Belgium), as detailed in Text S1 in the supplemental material.

### Microbiome sequencing.

We analyzed the microbiome interkingdom diversity of all samples using primers for bacteria (16S rRNA Illumina sequencing standard primers), archaea (16S rRNA; primers 787F/1000R), fungi (internal transcribed spacer [ITS]; primers ITS1/ITs2), the genus *Acanthamoeba* (18S rRNA; primers JDP1/JDP2) (49), and the class Heterolobosea (18S rRNA; primers Vahl730F_C/R-1200) (50) (Text S1). The Illumina libraries were prepared following the manufacturer’s instructions. High-throughput sequencing was performed with a MiSeq Illumina sequencer (2 × 300 bp) by the Biomics Pole (Institute Pasteur).

### Analysis of amplicon sequences.

Analysis of the microbiome data was done as detailed previously (51). Briefly, artefactual sequences, short reads (<50 bp), as well as sequences of low quality (quality score < 33) were discarded by using FASTX-Toolkit tools (http://hannonlab.cshl.edu/fastx_toolkit/index.html). The trimming of the sequences according to quality parameters was based on the PRINSEQ program (http://prinseq.sourceforge.net). Paired-end reads were joined by using the fastq-join script (https://expressionanalysis.github.io/ea-utils/). The Quantitative Insights into Microbial Ecology (QIIME) pipeline was used to discard chimeric sequences and to calculate the operational taxonomic units (OTUs) at 97% similarity. We selected the open-reference OTU picking method with the QIIME default taxonomy-training database. The taxonomic classification of the 16S rRNA and 18S rRNA reads was based on the Ribosomal Database Project (RDP) (52), and the ITS taxonomy was based on the Warcup ITS training set (53).

### Statistical analysis.

We estimated the microbial richness and diversity of the samples by calculating the total number of operational taxonomic units (OTUs), the Chao 1 richness estimator (54), and the Shannon diversity index (55). All analyses were based on the core diversity analysis script implemented in the QIIME pipeline (56). The 16S rRNA abundance table was rarefied at 60,000 reads per sample and the ITS abundance table was rarefied at 6,000 reads per sample, based on the sequencing effort. A hierarchical clustering analysis based on Bray-Curtis dissimilarity was applied to compare the microbial composition between samples. To statistically compare the diversity metrics between sample groups, we used the Wilcoxon signed-rank test implemented in the R program. Correlation analyses were based on the Pearson method. A multivariate analysis of variance based on dissimilarities (adonis test) to test the influence of external variables in explaining the differences in composition between different sample groups was used and was based on the Vegan package of the R program (57). Linear discriminant analysis (LDA) effect size (LEfSe) analysis was applied to identify the taxa characterizing the different sample groups (58).

### Quantification of bacterial load.

Quantitative real-time PCR (qPCR), based on the 16S rRNA gene, was performed to quantify the total bacteria in samples from patient A and used forward primer 520F (5′-AYTGGGYDTAAAGNG-3′) and reverse primer 802R (5′-TACNVGGGTATCTAATCC-3′). Standard curves were estimated by using serial 10-fold dilutions of purified and quantified amplicons. The PCR mixture was prepared in a final volume of 20 μl by adding 10 μl of 5× SYBR green qPCR master mix (Applied Biosystems), 0.8 μl of primers (10 mM), 3.4 μl of H_2_O, and 5 μl of the DNA sample. The amplification was performed on a Bio-Rad CFX qPCR instrument using the following program: 95°C for 3 min, followed by 40 cycles of 95°C for 5 s, 55°C for 30 s, and 72°C for 1 s. All reactions, including negative controls, were run in triplicate.

### Whole-genome sequencing.

To analyze the intrapatient evolution of L. pneumophila, 17 L. pneumophila isolates recovered from patient A (at days 4, 5, 10, 13, 14 [2 isolates], 16, 22, 26, 29, 30, 31, 33, 34, 36, 39, and 42) and 11 isolates recovered from patient B (at days 0 [6 isolates] and 69 [5 isolates]) were sequenced on an Illumina Nextseq500 (150-bp paired-end) machine, and Pacific Bioscience sequencing was performed by GATC Biotech. The genomes were analyzed as detailed in Text S1.

### Ethics approval and consent to participate.

The clinical sample collection of the Lyon University Hospital was declared to the French Ministry of Education and Research (number DC-2008-176), and written informed consent from the patients was obtained for this study.

### Availability of data.

All sequences have been entered in the European Bioinformatics Institute database under project accession number PRJEB33790.
