# Supplementary figures and images for: Persistent Legionnaires’ Disease and Associated Antibiotic Treatment Engender a Highly Disturbed Pulmonary Microbiome Enriched in Opportunistic Microorganisms
Source: mBio. 2020 May 19;11(3):e00889-20. doi: 10.1128/mBio.00889-20 (PMC7240155; doi:10.1128/mBio.00889-20)

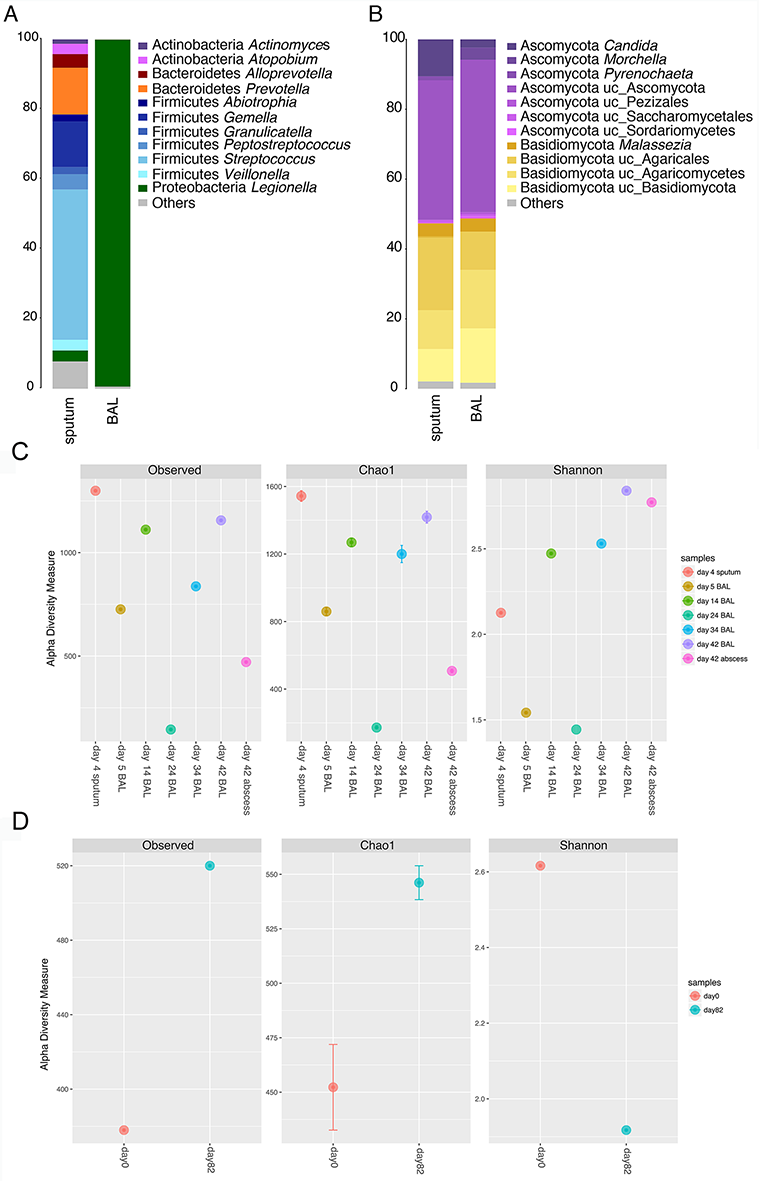

Supplement: FIG S1 [file mBio.00889-20-sf001.tif]

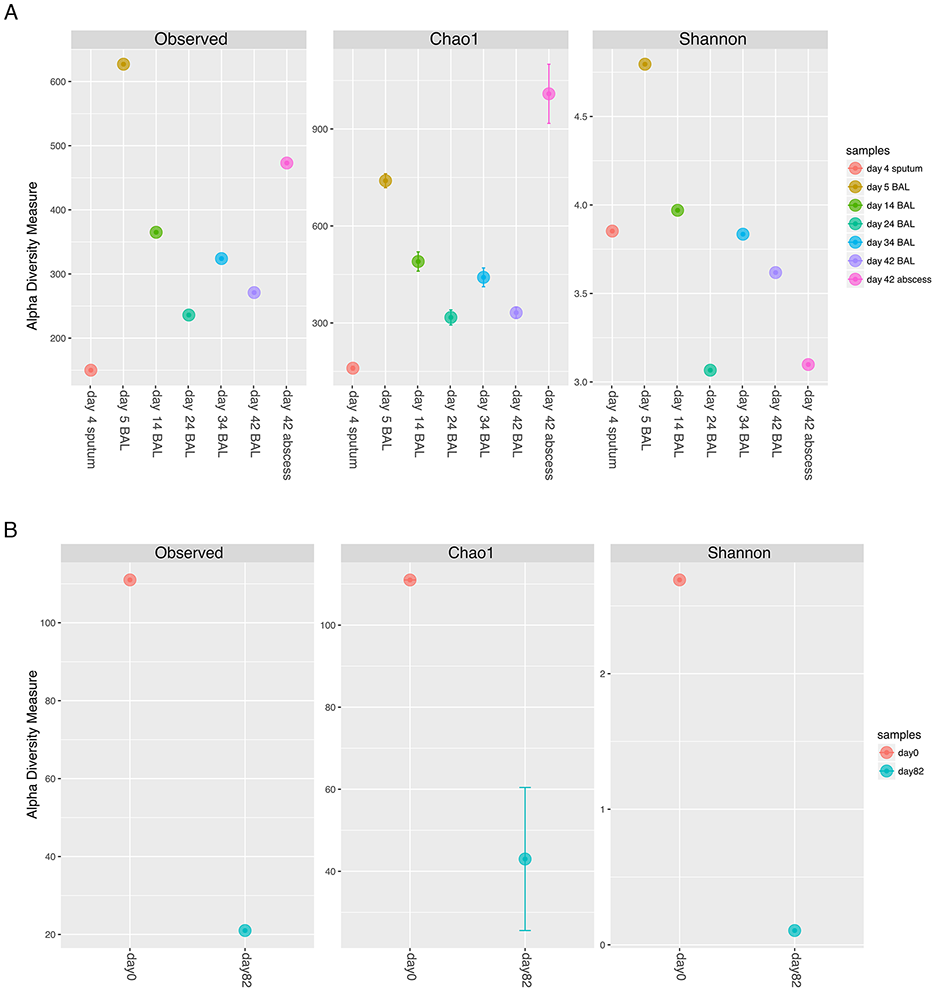

Supplement: FIG S2 [file mBio.00889-20-sf002.tif]

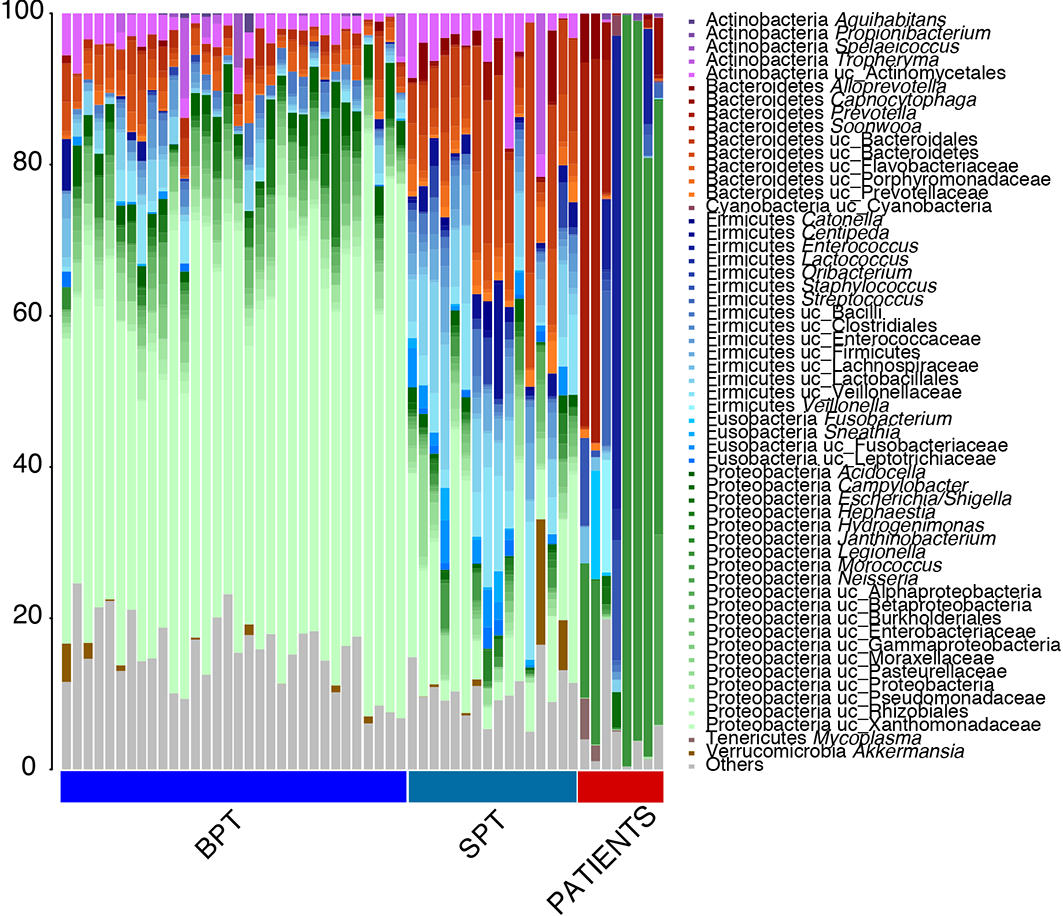

Supplement: FIG S3 [file mBio.00889-20-sf003.tif]

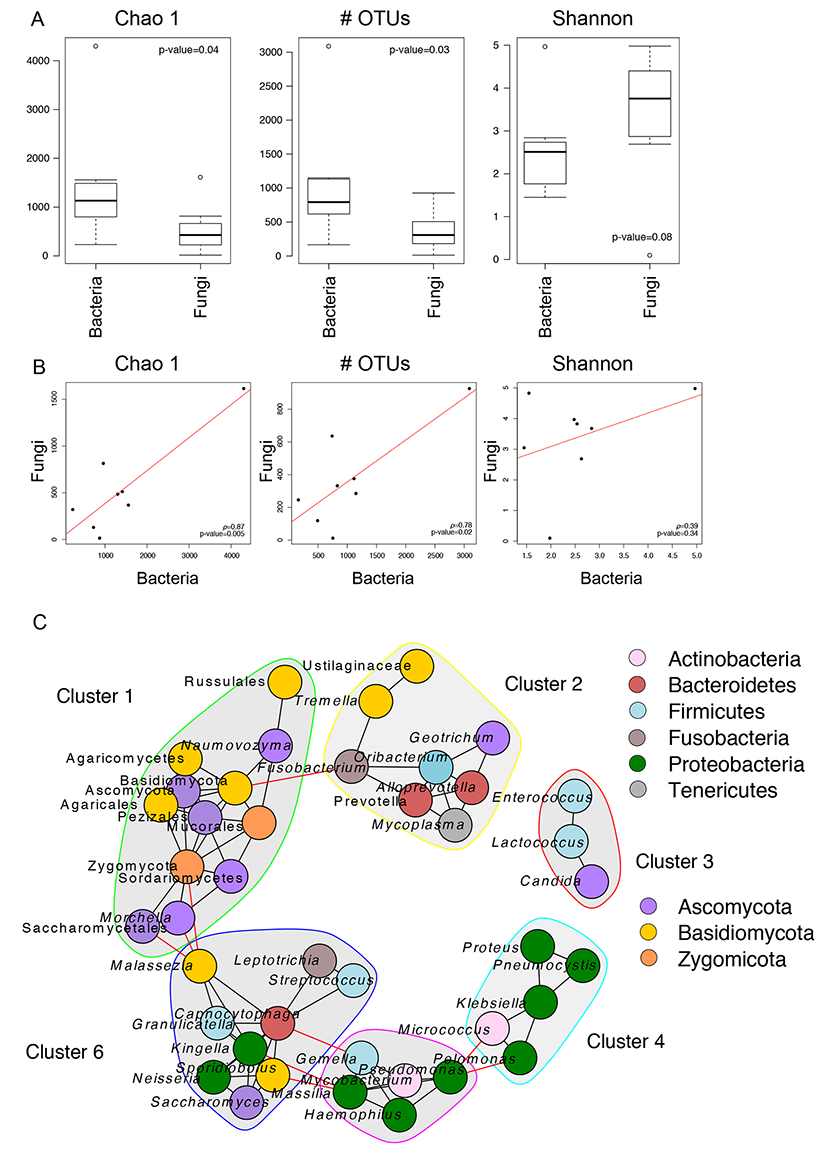

Supplement: FIG S4 [file mBio.00889-20-sf004.tif]

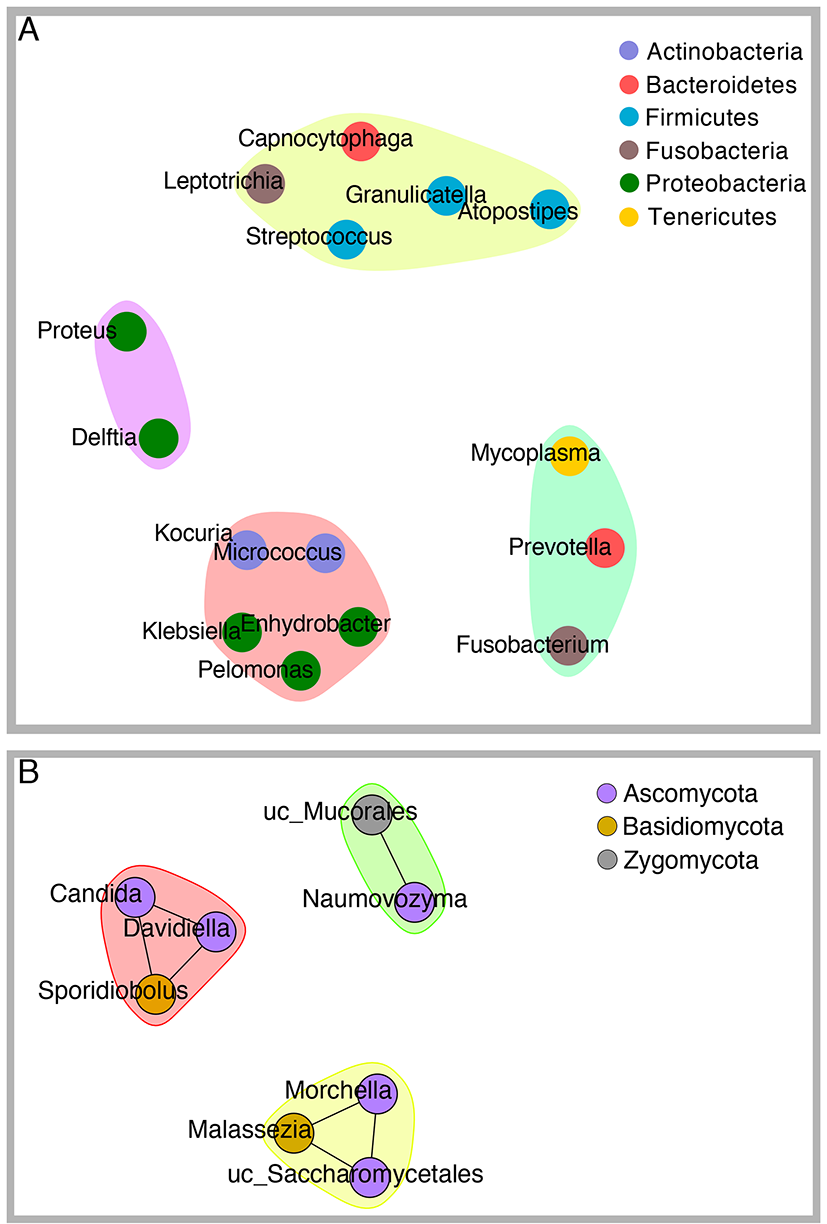

Supplement: FIG S5 [file mBio.00889-20-sf005.tif]
