# Supplementary material for: Persistent Legionnaires’ Disease and Associated Antibiotic Treatment Engender a Highly Disturbed Pulmonary Microbiome Enriched in Opportunistic Microorganisms
Source: mBio. 2020 May 19;11(3):e00889-20. doi: 10.1128/mBio.00889-20 (PMC7240155; doi:10.1128/mBio.00889-20)
Supplement: TABLE S1 [file mBio.00889-20-st001.docx]

**Supplementary Table 1A**. Proportion of reads classified as bacteria or archaea for each sample. The proportion of archaeal reads based on RDP taxonomic assignation considering a minimum criterium of bootstrap of 0.5 is also shown.

| **Sample** | **Bacteria (%)** | **Archaea (%)** | **Archaeal reads classified at bootstrap 0.5** |
| --- | --- | --- | --- |
| PatA-day5 | 27.3 | 72.7 | 6.7 |
| PatA-day14 | 44.1 | 55.9 | 2.7 |
| PatA-day24 | 36.3 | 63.7 | 12.6 |
| PatA-day33 | 34.8 | 65.2 | 10.7 |
| PatA-day42 | 98.3 | 1.7 | 97 |
| PatB-day0 | 89.9 | 10.1 | 89 |
| PatB-day82 | 92.3 | 7.7 | 91 |
| PatC-day0 | 35.9 | 64.1 | 52.4 |
| **Average** | **57.3** | **42.7** | **45.3** |

**Supplementary Table 1B**. Relative abundance of archaea present in the BAL samples. Classification of archaeal reads based on RDP taxonomy.

| Taxonomy | PatA5 | PatA14 | PatA24 | PatA33 | PatA42 | PatB0 | PatB82 | PatC0 |
| --- | --- | --- | --- | --- | --- | --- | --- | --- |
| Crenarchaeota;uc_Thermoproteaceae | 8.8 | 4.8 | 5.8 | 1.6 | 0 | 1.3 | 8.8 | 0 |
| Crenarchaeota;uc_Thermoproteales | 0.4 | 0.4 | 0 | 0 | 0 | 0 | 0.4 | 0 |
| Crenarchaeota;uc_Thermoprotei | 13.9 | 18.1 | 14.4 | 2.5 | 5.6 | 1.7 | 13.9 | 0 |
| Euryarchaeota;Methanobrevibacter | 73.8 | 51.5 | 56.9 | 70.7 | 55.6 | 95.6 | 73.8 | 100 |
| Euryarchaeota;Methanoregula | 0 | 4 | 0 | 0 | 0 | 0 | 0 | 0 |
| Euryarchaeota;uc_Euryarchaeota | 3.0 | 21.1 | 22.9 | 25.2 | 38.9 | 1.3 | 3.0 | 0 |

**Supplementary Table 1C**. Relative abundance of protozoa in the BAL samples (amoeba primers JDP1/JDP2). The classification is based on SILVA database (SILVA_132_QIIME_release).

| Taxonomy | PatA5 | PatA14 | PatA24 | PatA33 | PatA42 | PatB0 | PatB82 | PatC0 |
| --- | --- | --- | --- | --- | --- | --- | --- | --- |
| Eukaryota;uc_Acanthamoeba | 52.0 | 28.5 | 35.1 | 44.3 | 47.6 | 36.4 | 44.6 | 39.8 |
| Eukaryota;uc_Bilateria | 0.9 | 2.6 | 2.9 | 1.6 | 1.6 | 1.1 | 0.4 | 1.7 |
| Eukaryota;uc_Opisthokonta | 0.0 | 1.7 | 0.0 | 0.0 | 0.0 | 0.0 | 0.0 | 0.0 |
| Eukaryota;uc_Eukaryota | 47.1 | 67.1 | 62.0 | 54.1 | 50.7 | 62.5 | 55.1 | 58.5 |
