# Supplementary material for: Persistent Legionnaires’ Disease and Associated Antibiotic Treatment Engender a Highly Disturbed Pulmonary Microbiome Enriched in Opportunistic Microorganisms
Source: mBio. 2020 May 19;11(3):e00889-20. doi: 10.1128/mBio.00889-20 (PMC7240155; doi:10.1128/mBio.00889-20)
Supplement: TABLE S2 [file mBio.00889-20-st002.docx]

**Supplementary Table 2**. Relative abundance of eukaryotes in BAL samples from all the patients (amoeba primers Vahl730F_C/R-1200). The classification is based on SILVA database (SILVA_132_QIIME_release).

| Taxonomy | PatA5 | PatA14 | PatA24 | PatA33 | PatA42 | PatB0 | PatB82 | PatC0 |
| --- | --- | --- | --- | --- | --- | --- | --- | --- |
| Eukaryota;Trichomonas | 1.4E-02 | 0.3 | 89.4 | 70 | 0.7 | 66.7 | 88.1 | 0.9 |
| Eukaryota;uc_Bilateria | 0.6 | 0.7 | 0.1 | 0.2 | 6.8 | 0.5 | 0.9 | 4.3 |
| Eukaryota;uc_Eumetazoa | 0.1 | 0 | 0 | 3,8E-02 | 0.1 | 0 | 0.1 | 0.1 |
| Eukaryota;uc_Opisthokonta | 0.4 | 13.8 | 1.0 | 5.2 | 8.3 | 2.2 | 0.5 | 6.4 |
| Eukaryota;uc_Eukaryota | 64.9 | 85.0 | 9.4 | 24.2 | 84.2 | 30.5 | 10.4 | 88.3 |
| Unassigned | 34.0 | 0.1 | 0 | 0.3 | 0 | 0.1 | 0 | 0.1 |
