# Supplementary material for: Persistent Legionnaires’ Disease and Associated Antibiotic Treatment Engender a Highly Disturbed Pulmonary Microbiome Enriched in Opportunistic Microorganisms
Source: mBio. 2020 May 19;11(3):e00889-20. doi: 10.1128/mBio.00889-20 (PMC7240155; doi:10.1128/mBio.00889-20)
Supplement: TABLE S3 [file mBio.00889-20-st003.docx]

**Supplementary Table 3**. Bacterial families identified with statistically significantly different abundance between healthy and patient samples. Linear discriminant analysis (LDA) scores higher than 2 and p-values lower than 0.05 were consider as significant.

| Family | Sample type | LDA score | P-value |
| --- | --- | --- | --- |
| Xanthomonadaceae | healthy | 5.7 | 6.68E-06 |
| Veillonellaceae | healthy | 4.86 | 1.40E-03 |
| Flavobacteriaceae | healthy | 4.58 | 1.15E-02 |
| Acetobacteraceae | healthy | 4.52 | 1.17E-05 |
| Sphingomonadaceae | healthy | 4.28 | 2.22E-05 |
| Oxalobacteraceae | healthy | 4.24 | 1.57E-05 |
| Porphyromonadaceae | healthy | 4.14 | 1.58E-03 |
| Verrucomicrobiaceae | healthy | 4.12 | 2.21E-06 |
| Pseudomonadaceae | healthy | 4.08 | 6.46E-03 |
| Moraxellaceae | healthy | 4.07 | 2.42E-02 |
| Cellulomonadaceae | healthy | 4.03 | 1.95E-05 |
| Comamonadaceae | healthy | 3.86 | 1.14E-03 |
| Planctomycetaceae | healthy | 3.84 | 9.89E-06 |
| Sphingobacteriaceae | healthy | 3.83 | 5.46E-05 |
| Microbacteriaceae | healthy | 3.82 | 6.99E-05 |
| Chitinophagaceae | healthy | 3.76 | 8.87E-05 |
| Ruminococcaceae | healthy | 3.43 | 5.22E-04 |
| Cytophagaceae | healthy | 3.40 | 1.14E-04 |
| Burkholderiaceae | healthy | 3.38 | 1.40E-05 |
| Rhodobacteraceae | healthy | 3.34 | 1.72E-02 |
| Rhodocyclaceae | healthy | 3.30 | 3.26E-05 |
| Nocardiaceae | healthy | 3.23 | 2.53E-02 |
| Erythrobacteraceae | healthy | 3.17 | 7.75E-03 |
| Parachlamydiaceae | healthy | 3.17 | 1.64E-02 |
| Beijerinckiaceae | healthy | 3.16 | 1.23E-03 |
| Clostridiaceae | healthy | 3.10 | 2.75E-03 |
| Methylophilaceae | healthy | 3.09 | 2.12E-03 |
| Bdellovibrionaceae | healthy | 3.04 | 7.27E-03 |
| Bacteriovoracaceae | healthy | 3.03 | 1.13E-02 |
| Opitutaceae | healthy | 3.94 | 2.91E-03 |
| Rhodospirillaceae | healthy | 2.61 | 1.62E-02 |
| Oceanospirillaceae | healthy | 2.58 | 5.76E-04 |
| Gallionellaceae | healthy | 2.50 | 3.56E-03 |
| Micromonosporaceae | healthy | 2.48 | 2.45E-02 |
| Legionellaceae | pneumonia | 5.66 | 8.96E-14 |
| Staphylococcaceae | pneumonia | 4.63 | 4.67E-06 |
| Streptococcaceae | pneumonia | 4.59 | 8.71E-06 |
| Propionibacteriaceae | pneumonia | 3.49 | 2.39E-06 |
| Corynebacteriaceae | pneumonia | 3.44 | 2.02E-05 |
