# Supplementary material for: Persistent Legionnaires’ Disease and Associated Antibiotic Treatment Engender a Highly Disturbed Pulmonary Microbiome Enriched in Opportunistic Microorganisms
Source: mBio. 2020 May 19;11(3):e00889-20. doi: 10.1128/mBio.00889-20 (PMC7240155; doi:10.1128/mBio.00889-20)
Supplement: TEXT S1 [file mBio.00889-20-s0001.docx]

**Extended Material and Methods**

**Sample collection, *Legionella* detection and DNA extraction**.

For *Legionella* detection the PCRs were prepared in a final volume of 20 µL by adding the Master Mix 5X (TaqMan Probe LC2.0, Roche Diagnostics, France), 4 µL of primers and probe Lspp&Lp (R-DiaLegTM) or the internal control (DICD-YDL100) 2 µL, H2O (PCR grade) 2 µL and 10 µL of the extracted sample. The amplification was performed on a LC2 system using the following program: 95◦ C for 10 min followed by 45 cycles of 95◦C for 10 s, 60◦C for 40 s and 72◦C for 1 s and a final step of 30 s at 40◦C.

**Microbiome sequencing.**

For characterizing the bacterial fraction, the V3-V4 region of the 16S rRNA gene was amplified by PCR using the 16SrRNA Illumina sequencing standard primers with adapters. Forward primer (5'-CCTACGGGNGGCWGCAG-3') with adaptor (5'-TCGTCGGCAGCGTCAGATGTGTA TAAGAGACAG-3)' and reverse primer (5′- GACTACHVGGGTATCTAATCC-3') with adaptor (5'-GTCTCGTGGGCTCGGAGATG TGTATAAGAGACAG-3'). For each sample a 20 μl PCR mix was prepared, containing 5 μl of Buffer Taq (10X), 1 μl of 25 mM MgCl2, 0.5 μl of dNTPs (10 mM), 1.25 μl of each primer (10 mM), 0.25 μl of Phusion High-Fidelity DNA Polymerase (5u/μl), 0.5 μl of DMSO, 8.25 μl of nuclease-free water and 1 μl of DNA template. PCR conditions were: 95◦ C for 5 min followed by 25 cycles of 95◦C for 30 s, 55◦C for 1 min and 72◦C for 1 min and a final extension step of 7 minutes at 72◦C.

For characterizing the fungal composition, we amplified the ITS region by using the ITS1/ITs2 primers. Forward primer (5'-CTTGGTCATTTAGAGGAAGTAA-3') with adaptor (5'-TCGTCGGCAGCGTCAGATGTGTATAAGAGACAG-3') and reverse primer (5'-GCTGCGTTCTTCATCGATGC-3') with adaptor (5'-GTCTCGTGGGCTCGGAG ATGTGTATAAGAGACAG-3'). We used the same reaction mix and PCR conditions as described above, but 30 cycles. All the amplicons were checked by electrophoresis in agarose gel (1.4%).

To characterize the archaeal microbiome a commonly used region of the 16S rRNA to detect archaea was amplified by using the primers: 787F (5'-ATTAGATACCCSBGTAGTCC-3') with and 1000R (5’-GGCCATGCACYWCYTCTC-3'). The same sequencing adaptors and reaction mix described above were used for the PCR with the conditions: 95◦ C for 5 min followed by 30 cycles of 95◦C for 30 s, 62◦C for 1 min and 72◦C for 1 min and a final extension step of 7 minutes at 72◦C.

Two pair of primers to analyse the amoeba composition were: Primers JDP1 (5’-GGCCCAGATCGTTTACCGTGAA-3’) and JDP2 (5’- TCTCACAAGCTGCTAGGGAG TCA-3’), and Vahl730F_C (5’- TAATACTGCTGTAGTTAAAACGCCC-3’) and R-1200 (CCCGTGTTGAGTCAAATTAAGC). The same sequencing adaptors and reaction mix described above were used for the PCR with the conditions: 95◦ C for 5 min followed by 30 cycles of 95◦C for 30 s, 61◦C for 1 min and 72◦C for 1 min and a final extension step of 7 minutes at 72◦C for the primers 2F/12R. The same conditions were used for the other primers except for the annealing temperatures, being 63º and 57º for the JD1/JD2 and Vahl730F_C/ R-1200, respectively. For all PCR reactions negative controls were included and all amplicons of the samples and the controls of the kit were checked by agarose gel electrophoresis (1.4%).

**Whole-genome sequencing**

Reads were trimmed for low quality and adapter removing using trimmomatic 0.36 [3]. Trimmed reads were assembled using SPAdes [5]. Polymorphisms between isolates from the same Patient were searched by mapping the trimmed reads on the assembly of the 1st isolate using BWA v0.7.12 [6]. Pacbio sequencing was performed by GATC Biotech. Genomes were assembled using the SMRT Analysis software suite. Mutations in the genes rplD and rpIV, and in the domain V of 23S rRNA where mutations associated with macrolide resistances may occur, were analysed. Possible mutations associated to fluoroquinolone resistance in the genes gyrA, gyrB, and parC, and mutations conferring rifampicin resistance in the rpoB gene were searched. Trimmed reads were mapped on the different gene targets using BWA v0.7.12 and variant calling was done using the Naïve variant caller v0.0.4 [6].

**Ecological interactions between bacterial and fungal communities were observed in the human lungs**.

**Co-occurrence network analysis.**

We calculated co-occurrence networks for the 16SrRNA and the ITS OTU data to establish correlations. The networks were based on the OTU-rarefied abundance tables collapsed at genus level. We used SparCC to estimate the correlation coefficients between the genera, using 500 bootstraps from the genus-table to estimate the "two-sided" p-values [1]. The correlation matrix was filtered to keep significant correlations based on a p-value lower than 0.01 and converted in a network by using the igraph package implemented in R software [2]. The igraph package was used to display the network using a force-directed layout, representing the edges the correlation coefficients between taxa. We detected groups of taxa more connected between then and with fewer connections with other groups by using the Newman-Girvan algorithm also implemented in the igraph package.

Although the fungal community showed less changes than the bacterial community during antibiotic treatment, we identified some parallel changes in diversity between both groups (**Supplementary figures** **1**, **2**). To get insight into the ecology of these communities and their possible interactions, we used the Wilcoxon rank-test and compared the diversity metrics distribution of both communities (**Supplementary figure 5A**). As it seen in **Supplementary figure 5A** the bacterial fraction showed a significant higher species diversity richness (Chao 1 test and number of OTUs (p-value < 0.05)), but a more equitable distribution between the different species in the fungal community was observed (p-value < 0.05). Thus, the bacterial community is more variable in its composition and richer in species than the fungal community, but the distribution is less even probably due to the dominance of the pathogen and other opportunistic bacteria during infection and antibiotic therapy. To identify whether there might be a relation between the diversity of the bacterial and the fungal communities, Pearson correlation test comparisons between the alpha-diversity metrics were performed (**Supplementary figure 5B**). A significant positive correlation of the richness (number of OTUs and Chao 1) between bacteria and fungi was identified. The species distribution (based on Shannon Index) between both communities showed a positive trend but it was not significant. This suggests a cross-domain relation between both communities and a correspondence between different bacterial and fungal species, but the relation is not strongly dependent on their abundances.

Due to these results we established a correlation network for the microbiome and mycobiome and networks specific of each domain to identify putative associations between members of each community (**Supplementary figure 5**) (bacteria-fungi), **Supplementary figure 6A** (bacteria-bacteria) and **6B** (fungi-fungi)). Interestingly, we identified bacteria-bacteria, fungi-fungi and bacteria-fungi associations, however, the relationships within each domain were the most abundant (**Supplementary figure 5**). Furthermore, certain associations identified might be very strong, as they are maintained even when we analysed all possible interactions together (bacteria-fungi). Examples are the *Mycoplasma*-*Prevotella*-*Fusobacterium* (bacteria) (**Supplementary figure 6A**) or the *Morchella*-*Malassezia*-Saccharomycetales (fungi) cluster (**Supplementary figure 6B**).

We identified six main clusters in the mixed network. Cluster 1 was composed only of fungi belonging to three different phyla, whereas cluster 2 was composed of bacteria and fungi from different phyla. Most interestingly cluster 1 and 2 were connected through a predicted interaction between *Fusobacterium* and *Basidiomycota*. For cluster 1 a second interaction with cluster 6 containing fungi and bacteria was observed through interactions between *Saccharomyces*, *Zygomycota* and *Morchella* and *Malassezia* (**Supplementary figure 5C**). Cluster 6 is further connected with the bacterial cluster 5, which shows interactions with cluster 4 containing also only bacteria. These two bacterial clusters were enriched in Proteobacteria and Actinobacteria. Cluster 3 was composed of bacteria and fungi (*Enterococcus*-*Lactococcus*-*Candida)* and is the only one for which no interactions with other clusters of the network were predicted.

We observed a trend that bacteria and fungi from the same phyla clustered. This co-occurrence of phylogenetically closer related microorganisms may suggest cooperation and/or sharing the same niche instead of competition. Associations between bacteria and fungi have been described in the literature but little is known about their mechanisms of interaction. Similar to our results where an association in a co-occurrence network of *Fusobacterium* and *Prevotella* was observed such an interaction was also described for the lung microbiome of healthy individuals [[3]. Cluster 6 included the genera *Neisseria* and *Streptococcus,* both have recently been proposed as keystone species for the community structure of healthy neonatal airways [4].

Taken together, the microbial and the fungal community present in the lungs of humans are showing important interactions and seem to influence each other. *Malasezzia*, *Fusobacterium* or *Pseudomonas* seem to be key in the ecosystem since they are connecting different groups.

**References**

1. Friedman J, Alm EJ. Inferring correlation networks from genomic survey data. PLoS Comput Biol **2012**; 8(9): e1002687.

2. Csardi G, T. N. The igraph software package for complex network research. InterJournal, Complex Sy **2006**; 1695.

3. Segal LN, Clemente JC, Tsay JC, et al. Enrichment of the lung microbiome with oral taxa is associated with lung inflammation of a Th17 phenotype. Nat Microbiol **2016**; 1: 16031.

4. Pattaroni C, Watzenboeck ML, Schneidegger S, et al. Early-Life Formation of the Microbial and Immunological Environment of the Human Airways. Cell Host Microbe **2018**; 24(6): 857-65 e4.

**Supplementary Figure legends:**

**Supplementary figure S1**. **Comparison of the microbiome composition of BAL and sputum samples** **(patient A)** **and** **bacterial** **alpha-diversity of patients A and B**. **(A)** Bacterial composition of BAL and sputum of patient A. The taxonomy is based on the RDP **(B)** Fungal composition of BAL and sputum of patient A. The taxonomy is based on the Warcup ITS training set. The phyla and genera are shown for the most abundant groups (>1%). (**C**) Patient A, (**D**) Patient B show the bacterial diversities based on the 16SrRNA OTU-table. The diversity metrics Chao 1 richness estimator, number of OTUs and the Shannon Index were calculated. For each sample, it is shown the estimated values and the error bars, representing the range of the predicted values including the minimum and the maximum.

.

**Supplementary figure S2**. **Fungal alpha-diversity of patients A and B**. (**A**) Patient A, (**B**) Patient B. Fungal diversity was based on the ITS OTU-table. The diversity metrics Chao 1 richness estimator, number of OTUs and the Shannon Index were calculated. For each sample, it is shown the estimated values and the error bars, representing the range of the predicted values including the minimum and the maximum.

**Supplementary figure S3**. **Lung microbiome composition of healthy (SPT and BPT) and pneumonia samples**. The taxonomy is based on the RDP. The phyla and genera are shown for the most abundant groups (>2.5%).

**Supplementary figure S4**. **Correlation of diversity from bacterial and fungal communities in the lungs**. (**A**) Comparison of the diversity between fungal and bacteria of pneumonia samples. The diversity metrics Chao 1 richness estimator, number of OTUs and the Shannon Index were estimated for the two domains and statistically compared by using the Wilcoxon signed-ranked test. P-values lower than 0.05 were considered as significant. (**B**) Correlation of the Chao 1 richness estimator, the number of OTUs and the Shannon Index between the bacterial and fungal communities. The correlation is based on the Pearson method and p-values < 0.05 were considered as significant. (**C**) Co-occurrence network of bacterial and fungal communities. The network is based on the OTU-rarefied abundance tables collapsed at genus level. Only significant positive associations are shown (p-value < 0.01). The nodes represent the different taxa involved in the network and are coloured by phylum.

**Supplementary figure S5**. **Correlation networks of taxa in lung microbiome during pneumonia**. (**A**) Correlation of bacterial genera (**B**) Correlation of fungal genera. The networks are based on the OTU-rarefied abundance tables collapsed at genus level. Only significant positive associations are shown (p-value < 0.01). The nodes represent the different taxa involved in the networks and are coloured by phylum.
